# Nursing Activities Score at Discharge from the Intensive Care Unit Is Associated with Unplanned Readmission to the Intensive Care Unit

**DOI:** 10.3390/jcm11175203

**Published:** 2022-09-02

**Authors:** Junpei Haruna, Yoshiki Masuda, Hiroomi Tatsumi, Tomoko Sonoda

**Affiliations:** 1 Department of Intensive Care Medicine, School of Medicine, Sapporo Medical University, Sapporo 060-8556, Japan; 2 Department of Nursing, Tensei University, Sapporo 065-0013, Japan

**Keywords:** nursing activities score, unplanned ICU readmission, the stability and workload index for transfer

## Abstract

This study evaluated the accuracy of predicting unplanned the intensive care unit (ICU) readmission using the Nursing Activities Score (NAS) at ICU discharge based on nursing workloads, and compared it to the accuracy of the prediction made using the Stability and Workload Index for Transfer (SWIFT) score. Patients admitted to the ICU of Sapporo Medical University Hospital between April 2014 and December 2017 were included, and unplanned ICU readmissions were retrospectively evaluated using the SWIFT score and the NAS. Patient characteristics, such as age, sex, the Charlson Comorbidity Index, and sequential organ failure assessment score at ICU admission, were used as covariates, and logistic regression analysis was performed to calculate the odds ratios for the SWIFT score and NAS. Among 599 patients, 58 (9.7%) were unexpectedly readmitted to the ICU. The area under the receiver operating characteristic curve of NAS (0.78) was higher than that of the SWIFT score (0.68), and cutoff values were 21 for the SWIFT and 53 for the NAS. Multivariate analysis showed that the NAS was an independent predictor of unplanned ICU readmission. The NAS was superior to the SWIFT in predicting unplanned ICU readmission. NAS may be an adjunctive tool to predict unplanned ICU readmission.

## 1. Introduction

Unplanned readmission to the intensive care unit (ICU) is associated with unfavorable outcomes, such as poor prognosis [1] and prolonged ICU stay [2].

These adverse effects also have a negative impact on ICU bed utilization restrictions [3] and increased hospital costs [4]. Because unplanned ICU readmissions are related to hospital performance, increased hospital costs not only affect patients, but also impact the entire hospital system [5,6]. However, no methods have been set up to prevent ICU readmissions. Unplanned ICU readmissions occur in 4.5% to 9.2% of patients who are discharged from the ICU and are events that need to be prevented.

High severity of illness scores at ICU admission [7], the physiological indices at ICU discharge [8], and malignancy [9] are known risk factors associated with unplanned ICU readmission.

Various attempts have been made to predict unplanned ICU readmission. The aggregation of physiological indices, such as the National Early Warning Score [10] and the Stability and Workload Index for Transfer (SWIFT) [11] at the time of ICU discharge, can predict unplanned ICU readmission. However, factors that affect unplanned ICU readmission, and the validation of these tools for predicting unplanned ICU readmission, have not been fully elucidated.

The mortality rate and severity of illness in patients in the ICU are closely associated with the workload of the medical staff, including nurses. da Silva et al. [12] demonstrated that the nursing workload (NW) at the time of ICU discharge was associated with mortality and ICU readmission. NWs not only include nursing care, but also organ support status, and can comprehensively depict the patient’s condition. There are several scoring tools, such as the therapeutic intervention scoring system [13], nine equivalents of nursing manpower [14], and the Nursing Activity Score (NAS) [15], that can evaluate NWs in the ICU. The NAS has been widely used to evaluate the workload and allocation of medical resources in the ICU and to objectively measure the content of treatment [16]. Moreover, NAS has been independently associated with ICU readmission as a dichotomous variable [17]. However, there have been no comparisons between unplanned ICU readmissions using NWs and physiological indices. Since there are accurate predictive tools to prevent ICU readmission, we consider it meaningful to examine these from different perspectives. Therefore, we conducted this study to clarify the efficacy of predicting ICU readmissions using the NAS at ICU discharge compared to the prediction made by the SWIFT score.

## 2. Materials and Methods

### 2.1. Design, Setting, and Inclusion Criteria

This was a single-center, retrospective, observational study conducted at a university hospital. The study design and protocol were approved by the Institutional Review Board (IRB) of Sapporo Medical University (IRB-authorized number: 322-55, 9 September 2020). Patients with unplanned ICU admission during the period from January 2014 to December 2017 at the Sapporo Medical University Hospital were selected based on the electronic healthcare records (EHR). Among these patients, those who were discharged from the ICU and did not meet the exclusion criteria were enrolled.

Our hospital does not have a step-down unit. Rapid response teams have been established to facilitate early detection of patients who have a sudden deterioration in health.

Three researchers collected the data between August 2021 and September 2021. We initiated a post-ICU evaluation round to prevent ICU readmission in January 2018. We used data from 2014 to 2017 because of the potential influence of this post-ICU round on incidence of ICU readmission. ICU readmission was defined as readmission to the ICU within 7 days of ICU discharge, as previously described [18,19,20].

### 2.2. Exclusion Criteria

Patients who (1) were <18 years of age, (2) died in the ICU, (3) received a DNAR order during hospital stay, (4) were transferred to the different hospital within 7 days after ICU discharge, and (5) had missing electronic healthcare records data to calculate the SWIFT score and the NAS at the time of ICU discharge were excluded from this study.

### 2.3. Population

**Figure 1:**
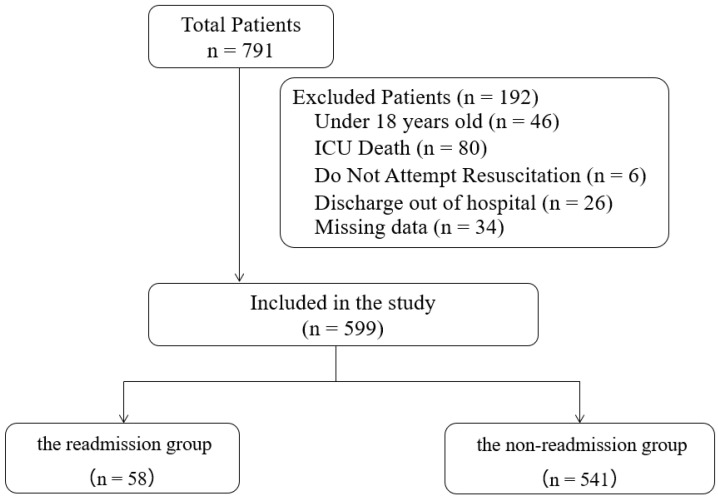
Patient enrolment flowchart.

Figure 1 shows a flow chart of patient enrollment. Among 791 patients who were inadvertently admitted to our ICU during the study period, 192 were excluded based on the exclusion criteria. The 34 patients for whom data on blood gas analysis were not collected within 24 h prior to ICU discharge were excluded. The remaining 599 patients were included in this study. A total of 58 patients (9.7%) were readmitted to the ICU within 7 days of ICU discharge.

### 2.4. Data Collection

The most recent patient data within 24 h before ICU discharge were collected from the EHR of our university hospital. Patient characteristics such as age, sex, and underlying disease were collected, and the Charlson comorbidity index (CCI), ventilator days, length of ICU stay, mortality, acute physiology and chronic health evaluation II (APACHE II) score, and sequential organ failure assessment (SOFA) score were calculated at ICU admission.

The variables of the SWIFT score at ICU discharge included the ICU admission source, length of ICU stay, the Glasgow Coma Scale score, PaO_2_/FIO_2_, and PaCO_2_ (Appendix A). The NAS was developed by Miranda et al. in 2003 to comprehensively describe nursing activities in the ICU beyond those related to the severity of illness [15]. The NAS represents approximately 80% of nursing activities in the ICU, which is more representative than the 43% obtained by the TISS-28 [15]. The NAS is an index comprising seven categories: basic activities, ventilatory, cardiovascular, renal, neurological, and metabolic support, and other specific interventions and 23 nursing activities, with weights ranging from 1.2 to 32 points (Appendix B). The NAS was calculated from the data collected according to the instruction manual [15].

### 2.5. Criteria for ICU Discharge

Intensivists, attending physicians, and ICU medical staff shared the status of a patient through rounds and meetings held every morning and discussed whether the patient was suitable for ICU discharge according to the guidelines for ICU admission, discharge, and triage [21]. In summary, the criteria for ICU discharge in our ICU were as follows.

Stable patients who did not require treatment or monitoring in the ICU.

Confirmation that the patient was ready for ICU discharge in a multidisciplinary conference with the attending physician.

### 2.6. Statistical Analysis

Data were assessed for Gaussian distribution using the Shapiro–Wilk normality test. Normally distributed data were presented as the mean ± standard deviation (SD), and non-normally distributed data were presented as the median and interquartile range (IQR). Categorical data were presented as counts and corresponding frequencies (%). Patient characteristics were analyzed using the Mann–Whitney U test for continuous variables and Fisher’s exact test for categorical variables. The SWIFT and the NAS were analyzed using the receiver operating characteristic (ROC) curves for each score and compared with the corresponding area under the receiver operating characteristic curve (AUROC). The cutoff value for predicting ICU readmission was calculated using the Youden index. We hypothesized that the NAS would be more useful than the SWIFT in predicting unplanned ICU readmission. To evaluate this hypothesis, logistic regression analysis was performed using unplanned ICU readmission as the dependent variable and SWIFT and the NAS as the independent variables. The SWIFT and the NAS were categorized by cutoff values in the ROC analysis and entered into the multivariate analysis. Variables that overlapped with the NAS and the SWIFT were not included in the multivariate analysis to avoid multicollinearity. Finally, age, sex, CCI, and SOFA scores upon ICU admission were selected as adjustment factors and entered into the multivariate analysis. CCI and SOFA were selected as covariates, because previous studies have reported their association with unplanned ICU readmission [22,23]. Statistical significance was set at *p* < 0.05. Statistical analyses were performed using SPSS software version 27 (IBM Corp., Armonk, NY, USA).

## 3. Results

### 3.1. Patient Characteristics

Patient characteristics are shown in Table 1. Sex, length of ICU stay, and the number of patients who received CRRT were significantly higher in the readmission compared to the non-readmission group. The SWIFT scores and the NAS in the readmission group were significantly higher than those in the non-readmission group.

### 3.2. Comparison of ROC Curves for Unplanned ICU Readmission between the SWIFT Score and NAS

**Figure 2:**
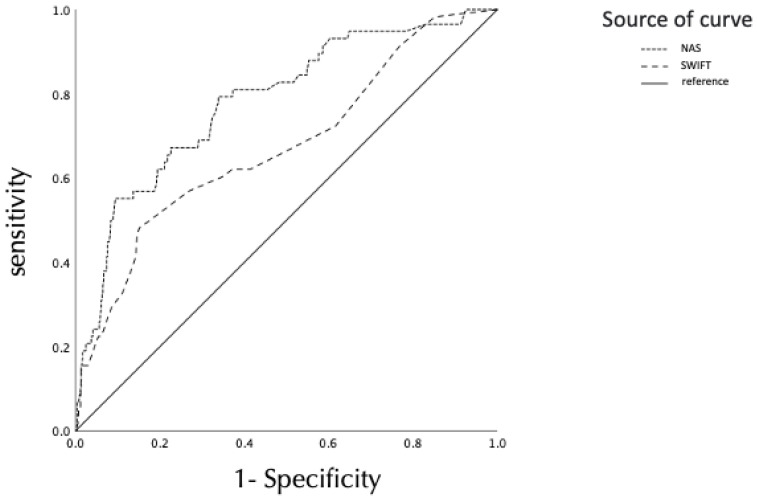
Area under the receiver operating characteristic curve for the NAS and SWIFT scores calculated at the time of intensive care unit discharge. NAS, Nursing Activities Score; SWIFT, Stability and Workload Index for Transfer.

The AUROCs of the SWIFT scores and the NAS are shown in Figure 2 and Table 2. The results of the ROC analysis showed that the optimal cutoff points of the SWIFT score and the NAS for predicting unplanned ICU readmission were 21 (sensitivity, 48%; specificity, 85%; AUROC, 0.68; 95% CI, 0.60–0.75) and 53 (sensitivity, 79%; specificity, 66%; AUROC, 0.78; 95% confidence interval (CI), 0.72–0.85), respectively.

### 3.3. Odds Ratio of the SWIFT Score and the NAS to Predict ICU Readmission

The results of the logistic regression analysis using the SWIFT score and the NAS are shown in Table 3. The odds ratio for predicting unplanned ICU readmission was 6.07-fold higher among patients with NAS > 53 than among those with NAS < 53 (95% CI, 3.04–12.1). In addition, the odds ratio for predicting unplanned ICU readmission was 3.61-fold higher among patients with SWIFT > 21 than among those with SWIFT < 21 (95% CI, 1.94–6.69).

### 3.4. Comparison of Each Item in the Nursing Activities Score

The comparison of each NAS item from the time of ICU discharge to 24 h prior to ICU discharge between the unplanned ICU readmission and the non-readmission group is shown in Table 4. The following components were significantly higher in the readmission than the non-readmission group: monitoring and titration, hygiene procedures, mobilization and positioning, support and care of relatives and patient, respiratory support, treatment for improving lung function, cardiovascular support, renal support, metabolic support, specific intervention(s) in the ICU, and specific interventions outside the ICU.

## 4. Discussion

Unplanned ICU readmission is associated with unfavorable outcomes [1] and prolonged hospital stay [2]. Severity scores [24] at ICU discharge and the National Early Warning Score [10] can predict unplanned ICU readmissions. Nursing care plays an important role in the management of critically ill patients. Traditionally, NW is associated with patient prognosis and severity. In this study, to clarify the relationship between NWs and unplanned ICU readmission, we compared the SWIFT score, which assesses unplanned ICU readmission, with the NAS at ICU discharge. The SWIFT score and the NAS in the readmission group were significantly higher than those in the non-readmission group. Moreover, the NAS within 24 h of ICU discharge was a better predictor of unplanned ICU readmission than SWIFT. Therefore, the NAS at ICU discharge may be a useful adjunctive tool for predicting unplanned ICU readmission.

There are several possible explanations for the superiority of the NAS at ICU discharge in predicting unplanned ICU readmission. A study showing an association between the mean NAS during ICU stay and ICU death reported the mean NAS to be 54.81 ± 2.34 points and that the risk of death increased with the NAS at ICU admission [25]. The cutoff value for the NAS in our study was 53 points, indicating that the NAS was as high as that reported for patients at high risk of ICU mortality. Although we did not calculate the NAS to determine ICU discharge, it was apparent that patients were discharged from the ICU with an unexpectedly high NAS. NW at ICU admission was correlated with the severity score [26]. However, the severity score has been reported to be more accurate than NW in predicting adverse events in the ICU [27]. ICU staff often use mechanical support, vital signs, and improved laboratory data as the basis for deciding to discharge patients from the ICU, and NW is rarely used as a criterion for ICU discharge. However, the results of this study suggest that NW may have an unexpectedly large impact on unplanned ICU readmission.

The NAS is a tool to evaluate nursing workload based on organ support as well as nursing care. Regarding the analysis of the categories of NAS, monitoring, hygiene procedures, mobilization and various organ supports were significantly higher in the readmission than in the non-readmission group. The risk factors reported for ICU readmission include ventilator support [3,9,28,29] and the need for pulmonary physical therapy [30,31,32] at ICU discharge. The most common reason for ICU readmission in this study was respiratory failure, which is similar to the results of previous studies [30,31]. Therefore, patients who require respiratory care at the time of ICU discharge require continuous pulmonary care for respiration in the general wards. When patients need pulmonary care at discharge from the ICU, we need to share information about their respiratory status with the medical staff in the general wards.

Patients who require more hours of patient care at ICU discharge are more frequently observed after ICU discharge due to readmission; however, in general wards, nurses must take care of multiple patients and cannot spend sufficient time on patient care, unlike in the ICU. Increased NW in general wards has been associated with an increased risk of pneumonia, urinary tract infections [33], nosocomial infections [34], and failure-to-rescue rates [35]. Furthermore, patient mortality increases as the number of patients cared for by each nurse increases [35]. ICU discharge with high NW may be linked to poor observation in general wards. Therefore, discharge from the ICU with a high NAS may increase the burden on nurses, and delays in the detection and appropriate treatment for complications may result in unplanned ICU readmission. In contrast, patients may be forcibly discharged from the ICU despite having a high NAS because of medical resource limitation issues, such as a limited number of ICU beds [21]. A follow-up system after ICU discharge reduced unplanned ICU readmission for patients who had to be discharged early from the ICU [36]. If patients at risk for unplanned ICU readmission are involuntarily discharged from the ICU, transition to a step-down unit, where nurses can observe them more frequently than general wards and provide adequate care including rehabilitation, should be considered. Moreover, considering the NAS as an adjunctive tool for the decision of ICU discharge may be useful in reducing unplanned ICU readmissions regarding the concern of the decreased quality of care in general wards.

Previous studies have reported that the SWIFT score is useful for predicting unplanned ICU readmission [37,38]. Kareliusson et al. showed that a SWIFT score ≥ 15 increased the risk of unplanned ICU readmission [38]. Gagic et al. also reported the usefulness of the SWIFT score vs. the APACHE III (AUROC of APACHE III vs. SWIFT; 0.62 vs. 0.75) [11]. In our study, the AUROC for the SWIFT score was 0.68, which was lower than that of NAS, suggesting that NAS at ICU discharge is superior to the SWIFT score for predicting unplanned ICU readmission. Similarly, Rosa et al. [23] concluded that the SWIFT score is not useful for predicting ICU readmission because of its low accuracy (AUROC, 0.65). However, the observation of physiological parameters is also important to assess the condition of critically ill patients. Although the SWIFT is less accurate than the NAS in predicting unplanned ICU readmission, we consider that comprehensive assessment, including physiological parameters and nursing WL, is necessary to predict unplanned ICU readmission.

This study has three limitations. First, this study was a retrospective observational study; thus, detailed information on the stability of chronic diseases at the time of ICU discharge was not available.

Second, this was a single-center, retrospective, observational study conducted at a university hospital. Therefore, whether the optimal cutoff point for NAS can be used to predict unplanned ICU readmission needs to be prospectively examined.

Third, when calculating the NAS, 23 items must be collected according to the NAS definition; therefore, the NAS may not be an easier-to-use triage tool than the SWIFT score for predicting ICU readmission. However, if a system such as the automatic calculation of the NAS by EHR can be developed, it may be possible to easily use the NAS to prevent unplanned ICU readmission. This study may provide a theoretical basis for future prospective investigations of whether the NAS can be used to prevent unplanned ICU readmissions.

## 5. Conclusions

We demonstrated the accuracy of the SWIFT score and the NAS for predicting unplanned ICU readmission. Our study showed that predicting availability of the NAS at the ICU discharge was superior to that of the SWIFT score. It may be useful to use the NAS at ICU discharge as an adjunctive tool for preventing unplanned ICU readmission.

## Figures and Tables

**Table 1:** Patient characteristics.

-	Readmission Group (n = 58)	Non-Readmission Group (n = 541)	*p*-Value
Age (years), Mean ± SD	64.3 ± 15.4	63.6 ± 16.1	0.74
Male, n (%)	43 (52.7)	313 (57.9)	0.02
Charlson Comorbidity Index, Mean ± SD	2.1 ± 1.9	1.6 ± 1.6	0.09
APACHE II, Mean ± SD	19.7 ± 7.2	18.7 ± 5.8	0.15
SOFA at ICU admission, Mean ± SD	6.1 ± 3.5	5.4 ± 3.2	0.10
ICU admission source			
Medical	46 (79.3)	324 (59.9)	0.004
Surgical	12 (20.7)	217 (40.1)	0.004
ICU discharge after hour	5 (8.6)	30 (5.5)	0.37
Reasons for ICU admission			
Sepsis, n (%)	15 (25.9)	99 (18.3)	0.16
Cardiovascular surgery, n (%)	5 (8.6)	85 (15.7)	0.18
Other surgery	5 (8.6)	88 (16.3)	0.18
Respiratory failure, n (%)	12 (20.7)	85 (15.7)	0.35
Circulatory failure, n (%)	2 (3.4)	73 (13.5)	0.02
Cerebrovascular disease, n (%)	7 (12.0)	63 (11.6)	0.83
Acute kidney injury, n (%)	3 (5.1)	17 (3.1)	0.43
Acute pancreatitis, n (%)	4 (6.9)	12 (2.2)	0.06
Endocrine disease, n (%)	1 (1.7)	10 (1.8)	1.00
Liver failure, n (%)	3 (5.2)	6 (1.1)	0.04
Metabolic disorder, n (%)	1 (1.7)	4 (0.7)	0.40
ICU length of stay (days), Mean ± SD	6.7 ± 7.4	4.5 ± 4.4	0.02
Mechanical ventilation, n (%)	35 (60.3)	303 (54.5)	0.05
Ventilator days, Mean ± SD	3.4 ± 4.2	2.1 ± 2.9	0.58
CRRT, n (%)	19 (32.8)	83 (15.3)	0.003
Mortality for 28 days	2 (3.4)	10 (1.8)	0.33
Reason for ICU readmission			
Respiratory failure	26 (44.8)	-	-
Circulatory failure	20 (34.5)	-	-
Renal failure	5 (8.6)	-	-
Electrolyte disturbances	5 (8.6)	-	-
Neurological failure	2 (3.4)	-	-
NAS and SWIFT score at ICU discharge			
NAS	22.9 ± 11.1	16.3 ± 7.6	<0.001
SWIFT	65.2 ± 15.7	49.2 ± 13.0	<0.001

Abbreviations: APACHE II, Acute Physiology and Chronic Health Evaluation II; SWIFT, Stability and Workload Index for Transfer; NAS, Nursing Activities Score; CCI, Charlson Comorbidity Index; SOFA, Sequential Organ Failure Assessment; CRRT, Continuous Renal Replacement Therapy.

**Table 2:** Comparison of predictive power and clinical utility of SWIFT and NAS of ICU readmission.

-	AUROC (95% CI)	*p*-Value	Cutoff	Sensitivity	Specificity
SWIFT	0.68 (0.60–0.75)	<0.001	21	0.48	0.85
NAS	0.78 (0.72–0.85)	<0.001	53	0.79	0.66

Abbreviations: SWIFT, Stability and Workload Index for Transfer; NAS, Nursing Activities Score; AUROC, Area Under the Receiver Operating Characteristic Curve; CI, confidence interval.

**Table 3:** Multivariate analysis of factors for prediction of unplanned ICU readmission.

-	Odds Ratio (95% CI)	*p*-Value
SOFA at ICU admission	0.82 (0.92–1.11)	0.824
Age	1.00 (0.97–1.02)	0.61
Men	1.81 (0.94–3.49)	0.08
CCI	1.26 (1.05–1.51)	0.013
SWIFT > 21	3.61 (1.94–6.69)	<0.001
NAS > 53	6.07 (3.04–12.1)	<0.001

Abbreviations: SWIFT, Stability and Workload Index for Transfer; NAS, Nursing Activities Score; CCI, Charlson Comorbidity Index; SOFA, Sequential Organ Failure Assessment; ICU, intensive care unit; CI, confidence interval.

**Table 4:** Comparison of NAS activities score items between the readmission group compared to the non-readmission group.

			Readmission Group (n = 58)		Non-Readmission Group (n = 541)		*p*-Value
Characteristic			N	%	N	%	
1	Monitoring and titration						
	1a Normal		20	34.5	392	72.5	<0.001
	1b More than normal		35	60.3	146	27.0	<0.001
	1c Much more than normal		3	5.2	3	0.6	0.014
2	Laboratory, biochemical and microbiological investigations		58	100.0	539	99.6	1
3	Medication, vasoactive drugs excluded		58	100.0	540	99.8	1
4	Hygiene procedures						
	4a Normal		16	27.6	413	76.3	<0.001
	4b More than normal		35	60.3	88	16.3	<0.001
	4c Much more than normal		0	0.0	2	0.4	1
5	Care of drains, all (except gastric tube)		33	56.9	290	53.6	0.679
6	Mobilization and positioning						
	6a Normal		14	24.1	246	45.5	0.002
	6b More than normal		36	62.1	221	40.9	0.003
	6c Much more than normal		6	10.3	8	1.5	0.001
7	Support and care of relatives and patient			0.0		0.0	
	7a About 1 h		11	19.0	26	4.8	<0.001
	7b About 3 h		1	1.7	0	0.0	0.097
8	Administrative and managerial tasks			0.0		0.0	
	8a Normal		57	98.3	536	99.1	0.459
	8b More than normal		1	1.7	10	1.8	1
	8c Much more than normal		0	0.0	0	0.0	1.000
Ventilatory support							
9	Respiratory support		53	91.4	398	73.6	0.002
10	Care of artificial airways		21	36.2	134	24.8	0.081
11	Treatment for improving lung function		28	48.3	149	27.5	0.002
Cardiovascular support				0.0		0.0	
12	Vasoactive medication		8	13.8	16	3.0	0.001
13	Intravenous replacement of large fluid losses.		0	0.0	0	0.0	1
14	Left atrium monitoring		6	10.3	1	0.2	0.005
15	Cardiopulmonary resuscitation after arrest, in the past period of 24 h		0	0.0	0	0.0	1
Renal support							
16	Hemofiltration techniques, dialysis techniques		10	17.2	28	5.2	0.002
17	Quantitative urine output measurement		55	94.8	514	95.0	1
Neurologic support							
18	Measurement of intracranial pressure		0	0.0	0	0.0	1
Metabolic support							
19	Treatment of complicated metabolic acidosis/alkalosis		0	0.0	0	0.0	1
20	Intravenous hyperalimentation		58	100.0	515	95.2	0.163
21	Enteral feeding through gastric tube or another gastrointestinal route		28	48.3	157	29.0	0.004
22	Specific intervention(s) in the ICU		5	8.6	9	1.7	0.007
23	Specific interventions outside the ICU		19	32.8	93	17.2	0.007

## Data Availability

Not applicable.

## Appendix A

**Table A1:** Stability and Work Load Index for Transfer.

Variable	Score
Original source of this ICU admission	
Emergency department	0
Transfer from a ward or outside hospital (any type of nursing care unit)	8
Total ICU length of stay (duration in days)	
<2	0
2–10	1
>10	14
Last measured PaO_2_/FIO_2_ ratio (during this ICU admission)	
>400	0
<400 and >150	5
<150 and >100	10
<100	13
Glasgow Coma Scale score at time of ICU discharge	
>14	0
11–14	6
8–10	14
<8	24
Last arterial blood gas PaCO_2_	
<45 mm Hg	0
>45 mm Hg	5

Abbreviations: ICU intensive care unit; FIO_2_ fraction of inspired oxygen; PaO_2_ partial pressure of arterial oxygen; PaCO_2_ partial pressure of arterial carbon dioxide.

## Appendix B

**Table A2:** Nursing Activities Score.

	Basic Activities	Score
1	Monitoring and titration	
1a	Hourly vital signs, regular registration and calculation of fluid balance	4.5
1b	Present at bedside and continuous observation or active for 2 h or more in any shift, for reasons of safety, severity, or therapy such as noninvasive mechanical ventilation, weaning procedures, restlessness, mental disorientation, prone position, donation procedures, preparation and administration of fluids or medication, assisting specific procedures	12.1
1c	Present at bedside and active for 4 h or more in any shift for reasons of safety, severity, or therapy such as those	19.6
	examples above (1b)	
2	Laboratory, biochemical and microbiological investigations	4.3
3	Medication, vasoactive drugs excluded	5.6
4	Hygiene procedures	
4a	Performing hygiene procedures such as dressing of wounds and intravascular catheters, changing linen, washing	4.1
	patient, incontinence, vomiting, burns, leaking wounds, complex surgical dressing with irrigation, and special procedures (e.g., barrier nursing, cross-infection related, room cleaning following infections, staff hygiene)	
4b	The performance of hygiene procedures took 2 h in any shift	16.5
4c	The performance of hygiene procedures took 4 h in any shift	20
5	Care of drains, all (except gastric tube)	1.8
6	Mobilization and positioning, including procedures such as: turning the patient; mobilization of the patient; moving from bed to chair; team lifting (e.g., immobile patient, traction, prone position)	
6a	Performing procedure(s) up to three times per 24 h	5.5
6b	Performing procedure(s) more frequently than 3 times per 24 h, or with two nurses, any frequency	12.4
6c	Performing procedure with three or more nurses, any frequency	17
7	Support and care of relatives and patient, including procedures such as telephone calls, interviews, counseling; often, the support and care of either relatives or the patient allow staff to continue with other nursing activities (e.g., communication with patients during hygiene procedures, communication with relatives while present at bedside, and observing the patient)	
7a	Support and care of either relatives or the patient requiring full dedication for about 1 h in any shift such as to explain the clinical condition, dealing with pain and distress, difficult family circumstances	4
7b	Support and care of either relatives or the patient requiring full dedication for 3 h or more in any shift such as death, demanding circumstances (e.g., large number of relatives, language problems, hostile relatives)	32
8	Administrative and managerial tasks	
8a	Performing routine tasks such as processing of clinical data, ordering examinations, professional exchange of information (e.g., ward rounds)	4.2
8b	Performing administrative and managerial tasks requiring full dedication for about 2 h in any shift such as research activities, protocols in use, admission and discharge procedures	23.2
8c	Performing administrative and managerial tasks requiring full dedication for about 4 h or more of the time in any shift such as death and organ donation procedures, coordination with other disciplines	30
Ventilatory support		
9	Respiratory support: any form of mechanical ventilation/assisted ventilation with or without positive end-expiratory pressure, with or without muscle relaxants, spontaneous breathing with or without positive end-expiratory pressure with or without endotracheal tube supplementary oxygen by any method	1.4
10	Care of artificial airways: endotracheal tube or tracheostomy cannula	1.8
11	Treatment for improving lung function: thorax physiotherapy, incentive spirometry, inhalation therapy, intratracheal suctioning	4.4
Cardiovascular support		
12	Vasoactive medication, disregard type and dose	1.2
13	Intravenous replacement of large fluid losses. Fluid administration 3 L/m^2^/day, irrespective of type of fluid administered	2.5
14	Left atrium monitoring: pulmonary artery catheter with or without cardiac output measurement	1.7
15	Cardiopulmonary resuscitation after arrest, in the past period of 24 h (single precordial thump not included)	7.1
Renal support		
16	Hemofiltration techniques, dialysis techniques	7.7
17	Quantitative urine output measurement (e.g., by indwelling urinary catheter)	7
Neurologic support		
18	Measurement of intracranial pressure	1.6
Metabolic support		
19	Treatment of complicated metabolic acidosis/alkalosis	1.3
20	Intravenous hyperalimentation	2.8
21	Enteral feeding through gastric tube or other gastrointestinal route (e.g., jejunostomy)	1.3
Specific interventions		
22	Specific intervention(s) in the intensive care unit: endotracheal intubation, insertion of pacemaker, cardioversion, endoscopies, emergency surgery in the previous 24 h, gastric lavage; routine interventions without direct consequences to the clinical condition of the patient, such as: radiographs, echography, electrocardiogram, dressings, or insertion of venous or arterial catheters, are not included	2.8
23	Specific interventions outside the intensive care unit: surgery or diagnostic procedures	1.9
